# Supplementary material for: The Divergent Roles of the Rice bcl-2 Associated Athanogene (BAG) Genes in Plant Development and Environmental Responses
Source: Plants (Basel). 2021 Oct 13;10(10):2169. doi: 10.3390/plants10102169 (PMC8538510; doi:10.3390/plants10102169)
Supplement: Supplementary file 1 [file plants-10-02169-s001.zip › Legends of the Additional Files S1-S12.pdf]

Additional File S1. Tissues and developmental stages throughout the life cycles of three rice varieties

Additional File S2. Signal intensities of the probe sets for the *OsBAG* and *OsCESA* families

Additional File S3. Primers used in this study

Additional File S4. Subcellular locations of OsBAGs proteins

Additional File S5. Chromosomal distribution of the *OsBAG* gene family

Additional File S6. Details of the 10 putative motifs

Additional File S7. Gene expression of Os*BAG1* and *3* revealed by qRT-PCR

Additional File S8. Top 100 co-expressed genes of the *OsBAGs* and *OsCESAs*

Additional File S9. Co-expression gene vicinity network for *OsCESAs*

Additional File S10. Promoter analysis of *OsBAGs*
The horizontal axis shows the six genes of *OsBAG*, and the vertical axis shows the corresponding cis- elements, the darker the color is the more cis- elements represented.

Additional File S11. Hierarchical cluster analysis of *OsBAGs* and *OsCESAs*

Additional File S12. Pearson correlation coefficient of *OsBAGs* and *OsCESAs* expression pattern
