# Supplementary figures and images for: The Divergent Roles of the Rice bcl-2 Associated Athanogene (BAG) Genes in Plant Development and Environmental Responses
Source: Plants (Basel). 2021 Oct 13;10(10):2169. doi: 10.3390/plants10102169 (PMC8538510; doi:10.3390/plants10102169)

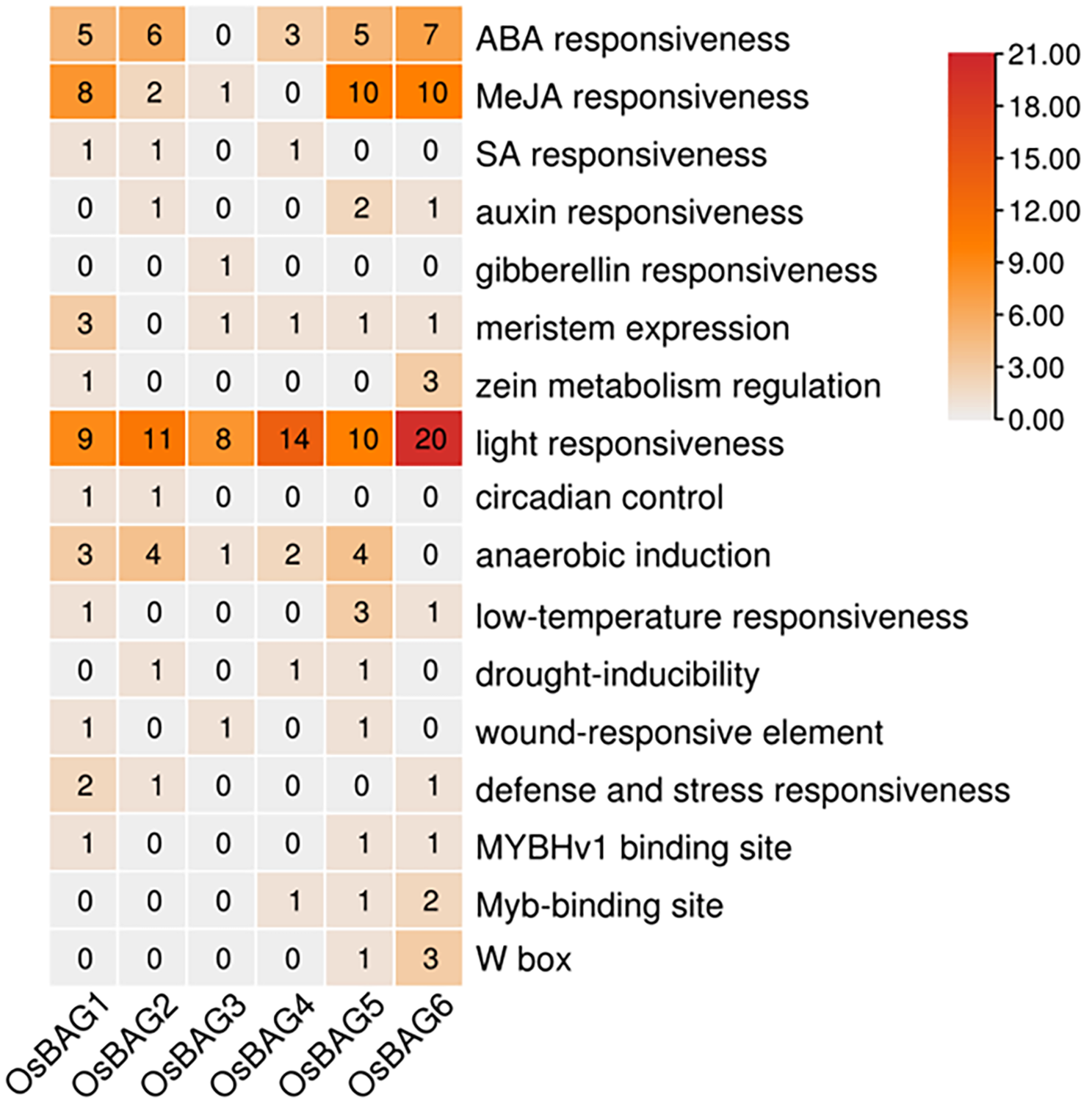

Supplement: Supplementary file 1 [file plants-10-02169-s001.zip › Additional File S10. Promoter analysis of OsBAGs.tif]

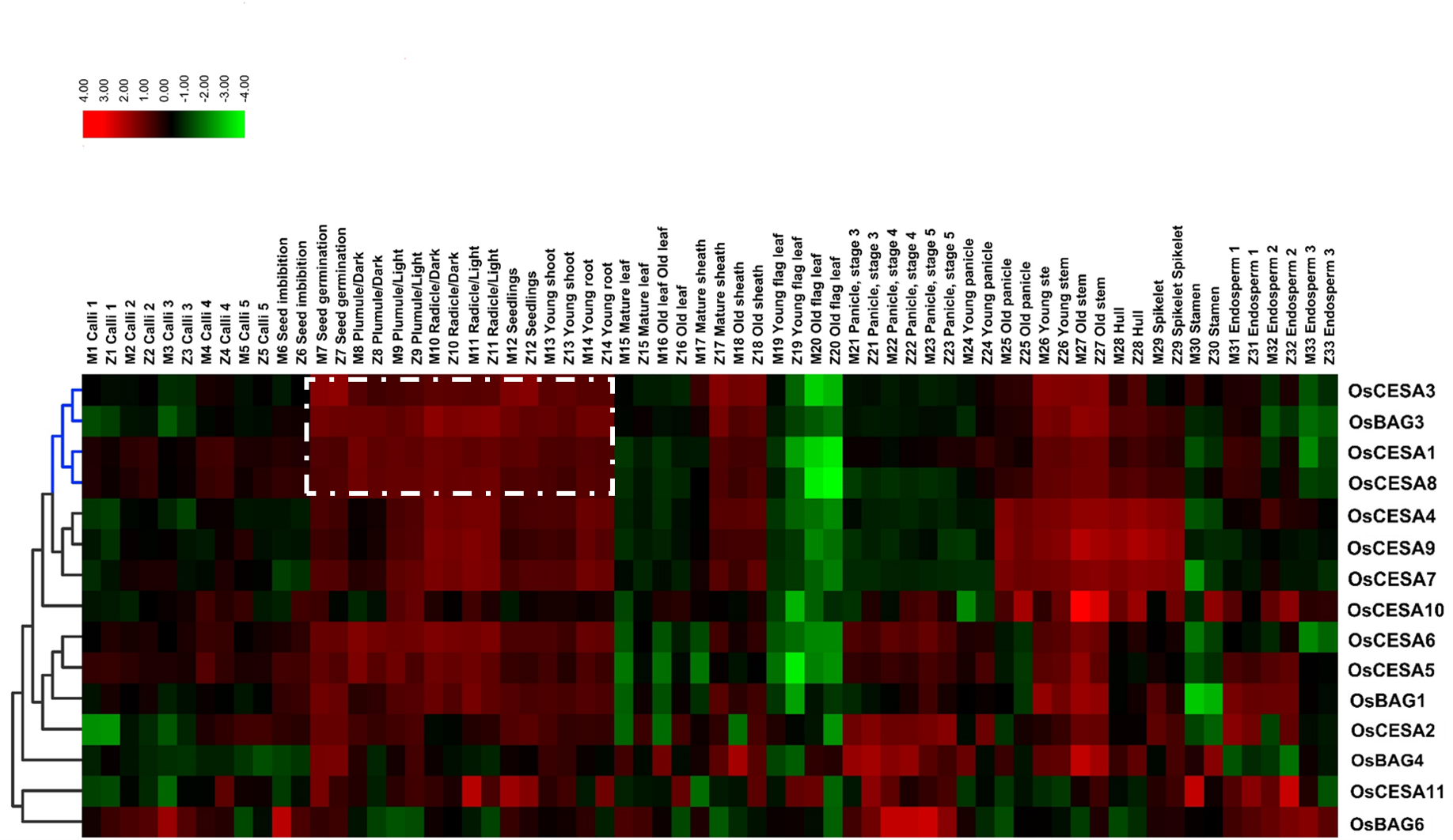

Supplement: Supplementary file 1 [file plants-10-02169-s001.zip › Additional File S11. Hierarchical cluster analysis of OsBAGs and OsCESAs.bmp]

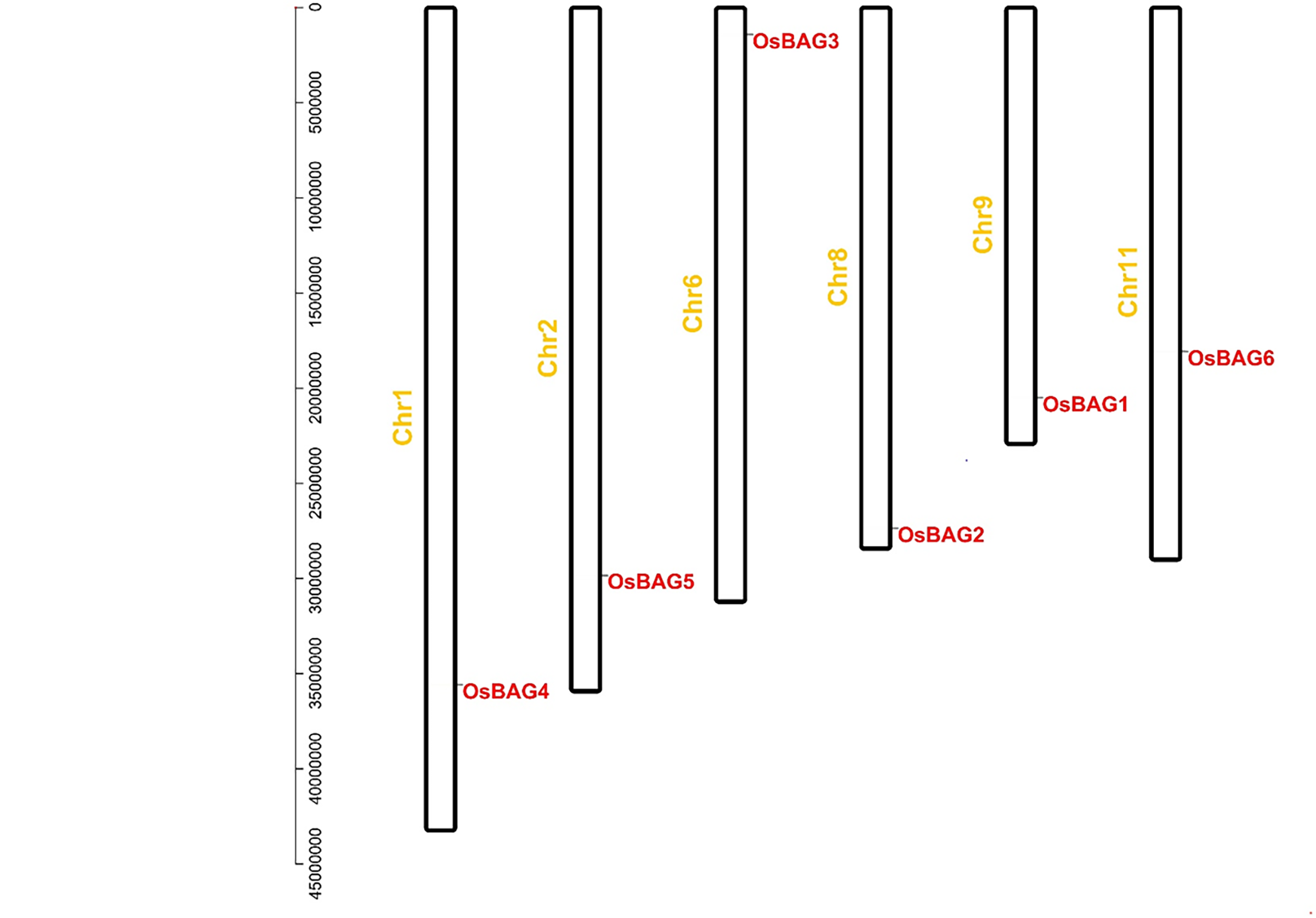

Supplement: Supplementary file 1 [file plants-10-02169-s001.zip › Additional File S5. Chromosomal distribution of the OsBAG gene family.tif]

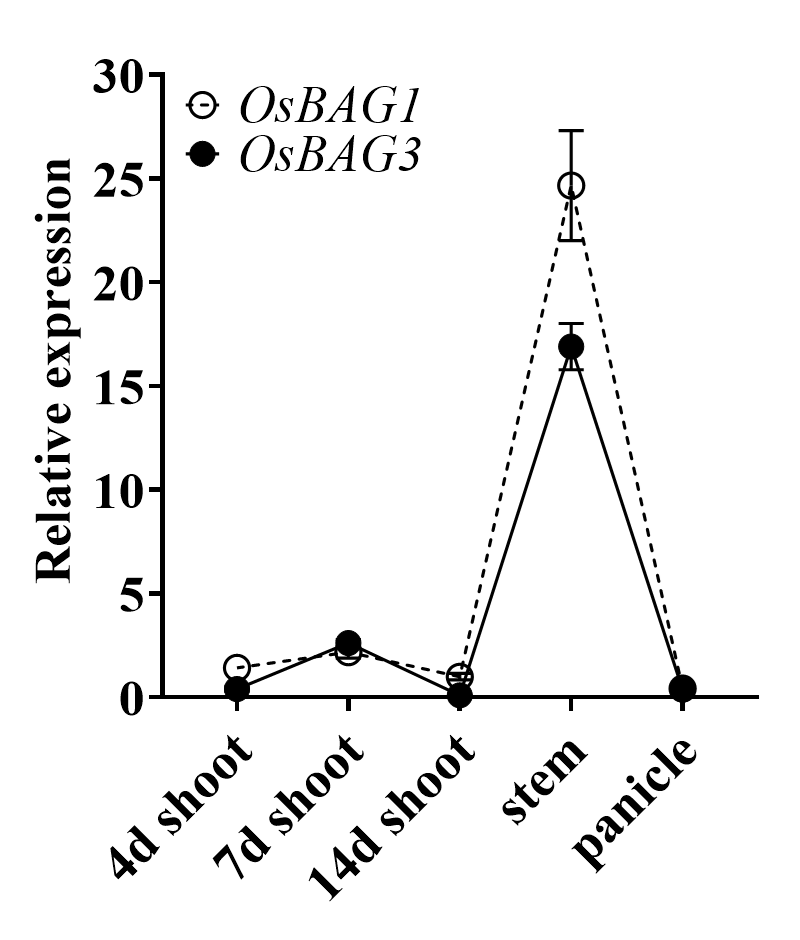

Supplement: Supplementary file 1 [file plants-10-02169-s001.zip › Additional File S7. Gene expression of OsBAG1 and 3 revealed by qRT-PCR.bmp]

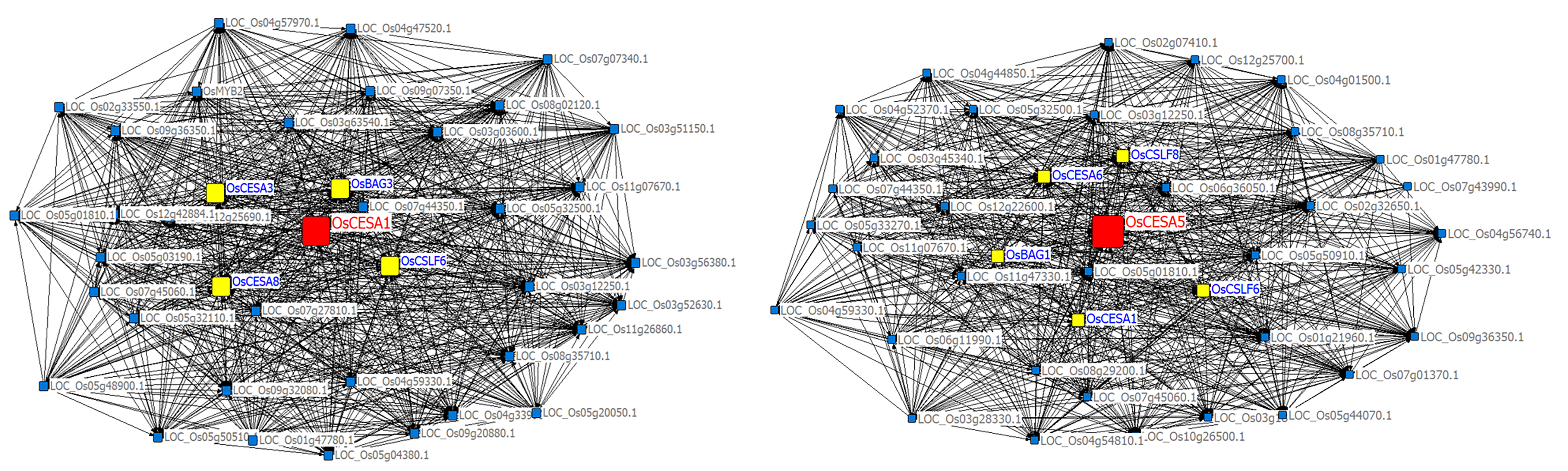

Supplement: Supplementary file 1 [file plants-10-02169-s001.zip › Additional File S9. Co-expression gene vicinity network for OsCESAs.tif]
